# Apparent age prediction from faces: A survey of modern approaches

**DOI:** 10.3389/fdata.2022.1025806

**Published:** 2022-10-26

**Authors:** Olatunbosun Agbo-Ajala, Serestina Viriri, Mustapha Oloko-Oba, Olufisayo Ekundayo, Reolyn Heymann

**Affiliations:** ^1^Computer Science Discipline, School of Mathematics, Statistics and Computer Science, University of KwaZulu-Natal, Durban, South Africa; ^2^Electrical and Electronic Engineering Science, University of Johannesburg, Johannesburg, South Africa

**Keywords:** apparent age, convolutional neural network, deep learning, facial aging, age prediction

## Abstract

Apparent age estimation *via* human face image has attracted increased attention due to its numerous real-world applications. Predicting the apparent age has been quite difficult for machines and humans. However, researchers have focused on machine estimation of “age as perceived” to a high level of accuracy. To further improve the performance of apparent age estimation from the facial image, researchers continue to examine different methods to enhance its results further. This paper presents a critical review of the modern approaches and techniques for the apparent age estimation task. We also present a comparative analysis of the performance of some of those approaches on the apparent facial aging benchmark. The study also highlights the strengths and weaknesses of each approach used for apparent age estimation to guide in choosing the appropriate algorithms for future work in the field. The work focuses on the most popular algorithms and those that appear to have been the most successful for apparent age estimation to improve on the existing state-of-the-art results. We based our evaluations on three facial aging datasets, including looking at people (LAP)-2015, LAP-2016, and APPA-REAL, the most popular and publicly available datasets benchmark for apparent age estimation.

## 1. Introduction

Age estimation is a very prolific area of research within the computer vision community (Huerta et al., 2015; Drobnyh and Polovinkin, 2017). There has been an increasing interest in age estimation from facial images (Drobnyh and Polovinkin, 2017) due to its increasing demands in various potential applications in security control (Abbas and Kareem, 2018), human-computer interaction (Abbas and Kareem, 2018), social media (Ruiz-Del-Solar et al., 2009), and forensic studies (Bouchrika et al., 2016). Although this subject has been extensively studied, the ability to estimate human ages reliably and correctly from face images is still far from satisfying human performance level (Onifade, 2015). There exist two kinds of facial age estimation: One is real (biological) age estimation, which determines the precise chronological or biological age of a person from the facial image (Shen et al., 2018); the other is apparent age estimation (Agustsson et al., 2017), which focuses on “how old does a person looks like” rather than predicting the real or biological age. The difference between the traditional real age estimation and apparent age estimation is that the age labels in apparent are annotated by human assessors rather than the real biological age. Some people may appear younger than their real age while others may appear older. As a result, the real age may differ from the apparent age of each subject.

**Figure 1:**
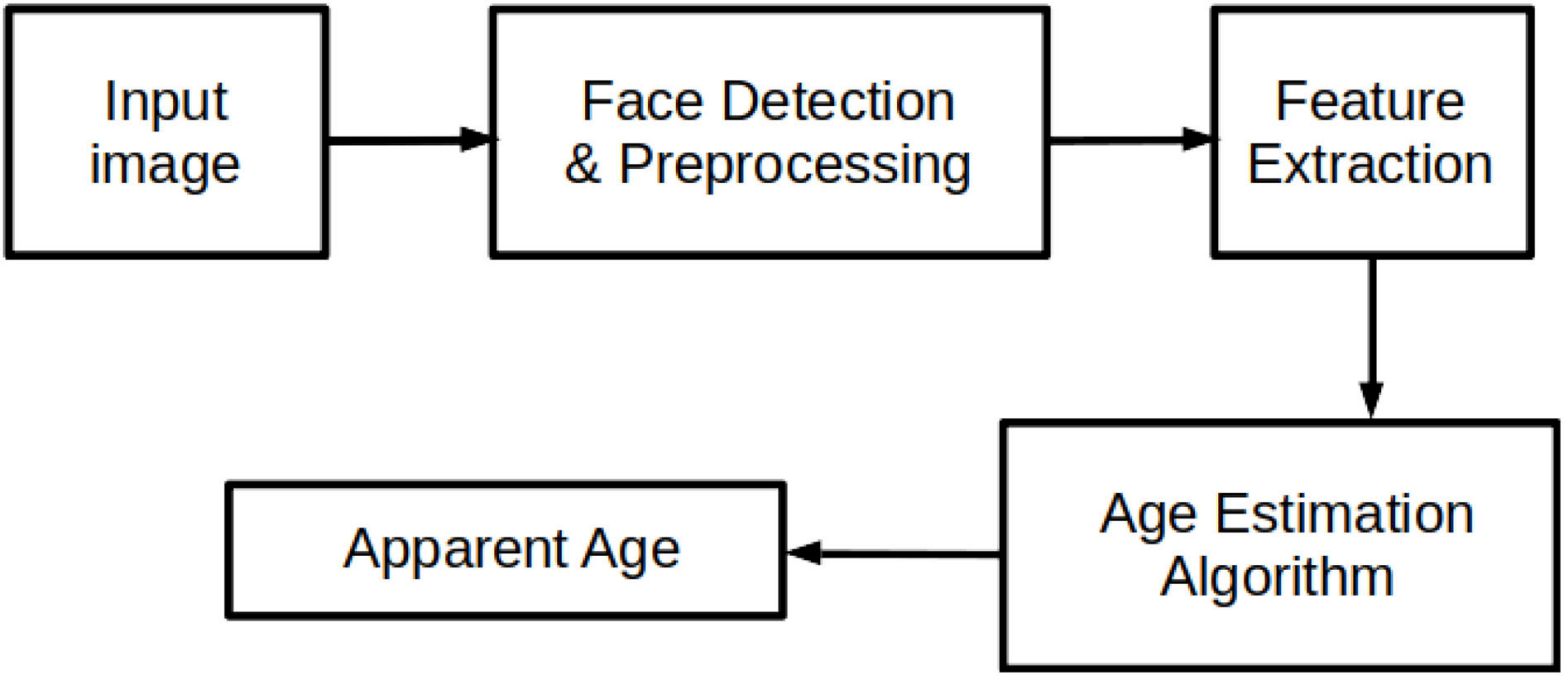
A typical apparent age estimation system. An age estimation system follows a general process that includes face detection, image preprocessing (landmark detection and face alignment), feature extraction (extracting the useful features from the input image), and classification itself.

Several methods have been proposed for apparent age estimation. The availability of huge data for training and an increase in computational power has made deep learning with convolutional neural network (CNN) a better method for the estimation task. Many researchers have studied several of these CNN methods, and these methods have improved the results and performances of apparent age estimation tasks. However, due to the challenging nature of apparent age estimation, further attempts to enhance the accuracy of the age estimation are still very much in progress. Researchers continue to examine different CNN and modern methods to enhance the results further. Hence, this paper critically reviews the modern approaches and techniques employed for apparent age estimation. We also present a comparative analysis of the performance of some of those approaches on standard apparent age datasets. The study also highlights the strengths and weaknesses of each approach to apparent age estimation to guide in choosing the appropriate approach to further improve the existing state-of-the-art results in the field. To ensure fairness in evaluating the performance of these approaches, we employed the popular apparent aging datasets and standard evaluation metrics that are widely used in literature in age estimation. Figure 1 displays the overall idea of a typical apparent age estimation system.

The contributions of this paper are highlighted as follows:

1. We outlined different state-of-the-art algorithms and techniques for apparent age estimation.
2. We described the performance evaluation analysis of different state-of-the-art models in apparent age estimation.
3. We presented three facial aging datasets widely employed in the research of apparent age estimation.
4. We also highlighted the standard performance evaluation metrics common in most literature for the apparent age estimation.

## 2. Application areas for apparent age estimation

Apparent age estimation has many notable real-world applications. Different intelligent application scenarios can benefit from computer-based systems that predict the apparent age of people among such application areas, including the following:

### 2.1. Medical diagnosis

Computer-based age prediction, as perceived by people, helps determine whether factors like the environment, depression, sickness, fatigue, and stress affect the premature aging of a person. This automatic age prediction will assist in obtaining the required information needed on a decision to improve the person's aging system (Escalera et al., 2015; Agustsson et al., 2017).

### 2.2. Effect of anti-aging treatment

Automatic apparent age estimation is also valuable for knowing the effect of some anti-aging treatments on people. The effectiveness of these anti-aging treatments, like topical treatment and hormone replacement therapy, can only be understood if an apparent age estimator is in place (Escalera et al., 2015; Rothe et al., 2018).

### 2.3. Facial beauty product development

The effect of some cosmetics products on facial beauty product development can only be discovered with an accurate apparent age predictor. It helps to bring customer insight, marketing story, and aesthetic experience to their product. The estimator assists in determining the best element for the formula's dispensing to deliver in the future for desirables and best products (Padme and Desai, 2015; Rothe et al., 2018).

### 2.4. Effect of plastic surgery

The essence of plastic surgery procedures is to reshape and restore the appearance of a person's body. The surgery is connected with beautification ideas, which should involve an extensive range of practical operations, including craniofacial surgery, reconstructive surgery, etc. However, to know the impact of plastic surgery procedures, there is a need for an automated system that determines “how old a person is like?” (Fu et al., 2010; Voelkle et al., 2012).

### 2.5. Movie role casting

An apparent age estimator also plays a role in selecting roles cast in movies, television programs, music videos, stage plays, video documentaries, and television advertisements, among others. In choosing a particular type of an actor, actress, singer, or dancer, for a specific role and character, the need to determine the person's age as perceived by people will be necessary (Padme and Desai, 2015; Rothe et al., 2018).

## 3. Description of apparent age estimation algorithms

**Figure 2:**
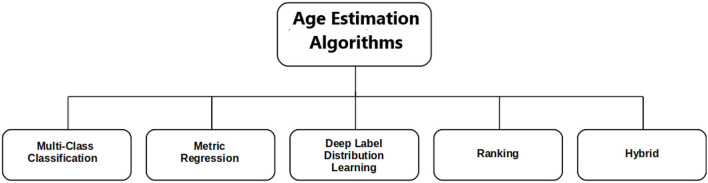
Classification of apparent age estimation approaches. The typical apparent age estimation methods are categorized into five different algorithms.

In this section, we present different algorithms and techniques used for apparent age estimation. As shown in Figure 2, most of these techniques fall into five different categories. Apparent age estimation can be modeled as a multi-class classification (MC), metric regression (MR), ranking, deep label distribution learning (DLDL), or a hybrid (combination of two or more techniques). We present a description of these algorithms and suggest the most effective approach in our opinion.

### 3.1. Multi-class classification

Multi-class classification approach views the ages or age groups category as an independent label and treats age value as a separate category and learns the age classifier to infer the person's age (Zhu et al., 2015; Malli et al., 2016; Feng et al., 2017). MC algorithm maximizes the probability of ground-truth class labels by not considering other classes. Nevertheless, the limited training samples and the class imbalance among most facial aging datasets can result in an overfitting problem (Gao et al., 2018).

### 3.2. Metric regression

The metric regression-based algorithm views the age class as a linearly progressing relationship and does not display the diversity of the aging method. It learns the trait most appropriate for mapping the age-value space from the feature space using the appropriate regularization method. Although, it is quite normal to address the age estimation task as an MR problem, which does minimize the mean absolute error (MAE) result and improves the performance of the estimation accuracy. However, MR generates an unsteady training mode, causing a significant error term affecting accuracy performance. Some of the typical regression methods include Gaussian Process (Zhang and Yeung, 2010), quadratic regression (Lanitis et al., 2004), and support vector regression (Guo et al., 2008).

### 3.3. Deep label distribution learning

Deep label distribution learning approach converts real-value age to a discrete-age distribution to fit the entire age distribution. It is an end-to-end learning model that solves the problem of insufficient training images experienced in most age estimation tasks. It relaxes the demand for a large number of training images and uneven data distribution by converting real age values to discrete age distribution to fit the whole age. The training instances connected with each class label will be increased without an increase in the number of training samples (Gao et al., 2018; Shen et al., 2018). However, it is usually observed that there is a lack of consistency between the employed evaluation metric and the training goals, generating an unsatisfactory result.

### 3.4. Ranking

The ranking-based algorithm uses age-axis tactics for age-class prediction and utilizes the relative order of the age. It uses relative age ranks instead of real age labels and ranks age class labels in descending order using their relevance to the presented face images to prevent making a decision for each age label that can simplify the problem (Chang et al., 2010; Li et al., 2012; Liu H. et al., 2017). Nonetheless, ranking algorithms can generate suboptimal results, especially when the training objectives and the evaluating metric are inconsistent.

### 3.5. Hybrid

The hybrid algorithm can be built by combining two or more algorithms in a parallel or hierarchical manner to produce a better performance. The algorithm makes the most of the advantage of the strengths of each algorithm to obtain a more robust system (Guo et al., 2008; Dib and El-saban, 2010; Choi et al., 2011). Unfortunately, combining two or more algorithms can result in a large storage overhead and computational costs, affecting its applicability in resource-constrained machines.

### 3.6. Summary of apparent age estimation algorithms

In this section, we summarized the main strengths and weaknesses of the different apparent age estimation algorithms in Table 1. Most of the existing state-of-the-art methods used MC and MR algorithms. The hybrid algorithm combines two or more algorithms, and this gives a better and more robust model compensating for the weak points in each algorithm with the strength of others. On the other hand, the ranking algorithm solves the problem peculiar to the classification algorithm by using the ordinal information of various ages and converting it into various binary classification problems. However, with DLDL, we obtained a better model by using the adjacent ages to generate label distribution for each age, even when the label distribution of the dataset is uneven. Table 2 presents the performance of state-of-the-art CNN architectures with a clear distinction of the best results on each dataset for apparent age estimation when evaluated using MAE and ϵ-error.

**Table 1:** Description of state-of-the-art algorithms in apparent age estimation.

**Algorithms**	**Description**	**Strengths**	**Weaknesses**
Multi-class classification (MC)	A multi-class classification method considers the age value as an independent category and later learns a classifier for the age classification task; It neglects the internal relationships between those age values.	•MC maximizes the expectation of the ground-truth set without considering other classes. •It also presents age value as a separate category and later learns a classifier for age estimation.	•MC algorithm can easily lead to over-fitting due to imbalances problem among the age classes and insufficient training images. •The method can also lead to unstable training.
Metric Regression (MR)	MR models the relationship between a set of features and a continuous target variable. It makes some predictions from data by learning the relationship between the features of the data and some continuous-valued answers.	•MR minimizes the MAE; The smaller the MAE, the better the performance of an estimator.	•Some outliers in the input data can cause a large error term, leading to an unstable training process and producing an unsatisfactory performance. •MR presents the age category as a linearly growing dependence rather than displaying the diversity of the aging process.
Deep Label Distribution Learning (DLDL)	DLDL is an end-to-end learning model that solves the problem of insufficient training images experienced in most age estimation tasks. It relaxes the demand for a large number of training images and uneven data distribution by converting real age values to discrete age distribution to fit the whole age distribution.	•DLDL overcome the uneven age label distribution problem by converting real age value to discrete age distribution to fit the whole age distribution. •DLDL also ease the necessity for a large number of images during training.	•DLDL may be suboptimal. •There might be an inconsistency during the training stage.
Ranking	Ranking uses the age-axis approach for age estimation. It employs the relative order of age to solve the classification bias problem caused by the dataset's unevenness of the dataset's sample images.	•The ranking model employs an age-axis approach that uses the relative order of age for the age classification. •The algorithm also transforms age estimation into a series of binary classification problems where the output of the rankers is aggregated directly from those binary outputs for the classification.	•Ranking produces inconsistency in the training objectives and evaluation metric. •Ranking method may be suboptimal at times.
Hybrid	A hybrid algorithm combines two or more modeling methods in a parallel or hierarchical manner to produce a better performance. It makes the most of the advantage of the strengths of each technique employed and is exacted to not only defeat other individual approaches but also make it robust.	•A hybrid model makes the most of the advantage of the strengths of each technique used, and it is expected to outperform other individual approaches. •Hybrid also produces a robust classifier.	•Combining two or more models might result in storage overhead and a huge computational cost. •It might be hard to deploy a hybrid model on resource-constrained devices.

**Table 2:** Description of state-of-the-art convolutional neural network (CNN) architectures in apparent age estimation.

**References**	**Approach**	**Pre-trained models**	**Algorithm**	**Dataset**	**External data**	**ϵ-Score**	**MAE**
Rothe et al. (2015)	20 CNN networks model	VGG	MC	LAP-2015	ImageNet; IMDb; WIKI;	0.278	3.221
Liu et al. (2015)	Real-value + Gaussian label distribution	GoogleNet	Hybrid	LAP-2015	CASIA-WebFace; CACD; WebFaceAge; MORPH-II;	0.2872	3.3345
Zhu et al. (2015)	Multiple models + RF + SVR	GoogLeNet	MR	LAP-2015	CASIA-WebFace; Adience; MORPH-II; FGNET; Lifespan; CACD; Private;	0.295	-
Ranjan et al. (2015)	DCNN-H-3NNR(Gaussian loss)	DCNN	MR	LAP-2015	CASIA-WebFace; Adience; MORPH-II;	0.373	-
Huo et al. (2016)	KL divergence + Softmax	VGG-16 + new CNN	DLDL	LAP-2015	MORPH-II; FG-Net; Adience; Web;	0.3057	-
Malli et al. (2016)	Ensemble of 3 CNNs	VGG-16	MC	LAP-2016	IMDb-WIKI;	0.3668	-
Antipov et al. (2016)	LDL + Classifcation	VGG-16	Hybrid	LAP-2016	IMDb-WIKI; Private;	0.241	-
Gurpinar et al. (2016)	VGG-Face + Kernel ELM	VGG-16	Hybrid	LAP-2016	-	0.3740	3.85
Liu W. et al. (2017)	GA-DFL + Multi-path CNN	VGG-16	MC	LAP-2015	-	0.369	4.21
Ranjan et al. (2017)	Euclidean + Gaussian loss functions	Novel CNN	MR	LAP-2015	MORPH-II; IMDb-WIKI; Adience;	0.293	-
Gao et al. (2017)	VGG-Face + DLDL(KL loss function)	ZF-Net + VGG-Net	DLDL	LAP-2015	-	0.31	3.51
Agustsson et al. (2017)	Residual DEX	VGG-16	MC	APPA-REAL	-	-	4.082
Gao et al. (2018)	LDL + Expectation Regression	ThinAgeNet	Hybrid	LAP-2015; LAP-2016;	-	0.272; 0.267	3.135; 3.452
Duan et al. (2018a)	CNN + ELM	AgeNet	Hybrid	LAP-2016	ImageNet; IMDb-WIKI; MORPH-II;	0.3679	-
Rothe et al. (2018)	DEX	VGG-16	MC	LAP-2015	ImageNet; IMDb-WIKI;	0.282	3.252
Li et al. (2019)	CNN + BridgeNet	VGGNet	MR	LAP-2015	-	0.26	-
Liu et al. (2019)	ODL (cross-entropy)	VGGNet	Ranking	LAP-2016	-	0.312	-
Deng et al. (2021)	CNN	VGG-16 + ResNet + GoogLeNet + AlexNet	Hybrid	LAP-2016	FGNET; MORPH	-	2.94; 2.97
Zhao et al. (2022)	adaptive mean residue loss	VGG-16 + ResNet50	DLDL	LAP-2016	FGNET	0.3882	3.61
Kjærran et al. (2021)	CNN	AgeNet	MC	APPA-REAL	UTK; IMDb	0.3882	3.61
Kjærran et al. (2021)	Gabor feature fusion + PCA + SVM + KPCA + CIAO-SA	Novel CNN	Hybrid	LAP-2016	Adience	-	2.10
Deng et al. (2021)	CNN	GenderNet; AgeNet; RaceNet	MR;Ranking	LAP-2016	MORPH2; FGNET	-	2.47; 2.59; 2.67

## 4. Performance evaluation analysis of state-of-the-art methods in apparent age estimation

Apparent age is “how old a person looks like”? A significant amount of study has been done to extract facial features from faces to determine the apparent age of people. In Rothe et al. (2015) developed a classification-based solution [deep expectation (DEX)] for apparent age. The authors used the VGG-16 architecture that was initially pre-trained on ImageNet before further fine-tuning the newly collected IMDb-WIKI dataset of 500,000 faces of unconstrained images. The CNN-based model was addressed as a deep classification problem. As part of the solution, they employed an open source face detector by Mathias et al. (2014) to locate the face in an image before extracting the CNN predictions from an ensemble of 20 networks on the cropped face which was also fine-tuned on the LAP-2015 dataset. The developed model achieved a great result but demanded more computational cost and large storage overhead to pre-trained the model on huge datasets like ImageNet and IMDb-WIKI.

Liu et al. (2015) later presented a hybrid model (AgeNet) that fuses a regression (real-value) and classification (Gaussian label distribution) to solve the apparent age estimation task. The two models employed GoogleNet CNN to learn informative age representations after preprocessing the images using face detection, facial landmark localization, and face normalization. The models were initially pre-trained on a large-scale facial aging dataset with identity labels and then fine-tuned on another large-scale age dataset with unconstrained age label before it was fine-tuned on the training images of the original LAP-2015 with apparent age labels. The hybridized model achieved a second place position in the 2015 edition of the Chalearn Looking At People (LAP) competition. However, the employed GoogleNet 22-layers deep convolution neural network was too deep to be implemented on resource-constrained devices.

Zhu et al. (2015) studied a method that utilized the deep representations trained in a cascaded way. The approach also employed GoogleNet design, initially pre-trained with face images without age labels, then on data with chronological age labels to fine-tune the network parameters before finally fine-tuning the apparent age model on the apparent age dataset itself. The proposed approach consists of four different processes: an image pre-processing stage (face detection and landmark localization), a CNN design architecture in a cascade way, a coarse-to-fine design consisting of age grouping and local age estimators, and fused predictors. Although the model achieved an ϵ-error of 0.2948 on the Chalearn LAP-2015 dataset with a third position at the 2015 edition of ChaLearn LAP challenge, it demands more computational cost and overhead to pre-train very large datasets on equally large CNN architecture.

In Ranjan et al. (2015), developed a regression-based automatic age estimation model. The approach estimates apparent age from unconstrained images using deep CNN (DCNN). The architecture consists of four steps: face detection, face alignment, feature extraction, and a 3-layer neural network regression. They employed a deep pyramid deformable part designed for the face detection phase and an ensemble of regression trees method (dlib C++ library) for the face alignment phase. However, for the feature extraction phase, they proposed a method that obtained traits from the pool5 layer of a pre-trained DCNN model without needing to re-tune the pre-trained DCNN network for face description tasks on age estimation data. For the age estimation phase, they used a 3-layer neural network regression model with the Gaussian loss function and a hierarchical learning approach to further boost the result of their work. Consequently, their approach achieved comparable results when evaluated on the LAP-2015 dataset. However, some outliers in the input data can cause a large error term, leading to an unstable training process.

Huo et al. (2016) introduced a deep CNN with distribution-based loss functions. The distributions utilized the ambiguity induced *via* manual labeling by learning a better model rather than using ages as the target. The method employed two different types of deep CNN models with different architectures: the first is based on the popular pre-trained VGG-16 CNN; the second is based on a different CNN architecture. The VGG-16 architecture is fine-tuned on three different datasets before finally fine-tuning the two models on the competition dataset. The fusion of the two outputs generated the final predicted ages. The method achieved an ϵ-error of 0.3057 when evaluated on the LAP-2015 dataset. However, a distribution-based loss function might yield an inconsistent result during the training stage.

Malli et al. (2016) investigated an approach that used VGG-16 deep CNN models pre-trained on the IMDb-WIKI dataset and fine-tuned on the same dataset. The approach, an ensemble of deep learning methods, extracted deep features from the 7th layer (FC) of the VGG-FACE model and trained a 3-layer neural network with two hidden layers using deep features. They treated age estimation as a classification problem; as such, they assigned age labels within standard deviation boundaries as true. The approach significantly improved after fine-tuning the VGG-16 models for testing the age-shifted grouping technique. Although the designed fusion schemes with an ensemble of deep learning methods achieved a ϵ-error of 0.3668 on the test set of the LAP-2016 dataset when evaluated, it can lead to overfitting if an appropriate regularization algorithm is not utilized.

In Antipov et al. (2016), the authors proposed a pre-trained VGG-16 CNN model that combined two separate models: the general and the children. The general model was initially trained on the huge IMDb-WIKI dataset for biological age estimation and then fine-tuned for the apparent age estimation task. The children model used a pre-trained VGG-16 network and trained the children (private dataset of children between 0 and 12 years old) dataset before it was also fine-tuned on the original apparent age estimation dataset. The children's network was fine-tuned from the general network. The method involves using separate age encoding strategies for training the general and children networks; a label distribution encoding for the general network and 0/1 classification encoding for the children network. The hybridized approach combines the strength of the two algorithms hierarchically but employs a more lighter CNN architecture that allows its applicability on a resource-constrained device.

Gurpinar et al. (2016) proposed a two-level method for apparent age estimation of facial images. They classified samples into overlapping age groups, and within each age group, apparent age is estimated with local regressors before fusing the output for the final age estimation task. The method involved three phases: face alignment, feature extraction, and model learning. They used a deformable parts model (DPM) for the face detector and a pre-trained CNN for feature extraction from already aligned images before employing Kernel extreme learning machines for classification. The method's effectiveness was evaluated on the ChaLearn LAP 2016 dataset, and they reported an ϵ-error of 0.374 on the test set and MAE of 3.85.

Liu W. et al. (2017) proposed a group-aware deep feature learning (GA-DFL) technique for apparent age estimation. The CNN-based method acquired the needed feature descriptor directly from raw pixels of the face images. The ordinal ages were split into sets of discrete collections to learn deep feature transformations. They also designed a multi-path CNN approach to combine the corresponding information. The experimental results proved that the approach produced an excellent performance when compared with state-of-the-art methods. It was evaluated on three known face aging datasets and obtained an MAE of 3.93 (FG-NET), 3.25 (MORPH-II), and 4.21 (LAP-2015) with an error of 0.369.

Ranjan et al. (2017) presented a novel multi-purpose CNN model that concurrently solves the problem of apparent age estimation, genders recognition, face detection, pose estimation, landmark localization, smile detection, face verification, and recognition from any unconstrained face image. The approach, an improvement of the work in Ranjan et al. (2015), was trained in an MTL framework that develops a synergy among several face-related tasks improving the performance of each of those tasks through learning robust features for the distinct tasks. They employed multiple tasks enabling the network to learn the correlations between data from many distributions in an efficient way. They used Chalearn LAP-2015 and FG-NET datasets to model the age classifier, and it achieved a comparable result when it was evaluated on the same datasets.

Gao et al. (2017) also proposed a DLDL approach, an end-to-end deep learning design that employed label ambiguity in both feature and classifier learning. The approach prevented the network from overfitting even when an inadequate training dataset was used. They converted the label of each image into a discrete label distribution and learned the label distribution by minimizing a Kullback-Leibler divergence between the ground-truth and predicted label distributions using deep ConvNets. This is necessary to resolve the problem of ambiguous information among labels. Extensive experimental results revealed that the proposed approach for apparent age estimation performed significantly better than state-of-the-art methods on the same task when evaluated on MORPH-II and LAP-2015 datasets.

Agustsson et al. (2017) proposed a model called Residual DEX, an enhancement of DEX (Rothe et al., 2015). The apparent age model was addressed as a classification problem considering the age value as an independent category. The idea of residual is that the error between the ground truth labels and the rough DEX estimation can be tackled with a unique model. The model learns a new regressor with the same CNN architecture in Rothe et al. (2015) to predict DEX residuals, and this significantly contributed to the performance of the new apparent age estimator.

The authors in Gao et al. (2018) designed a lightweight CNN architecture that collectively learned age distribution and regressed it. The CNN-based approach, ThinAgeNet, employed the compression rate of 0.5. The network was further trained on a quite small model with a compression rate of 0.25 and called it TinyAgeNet. The method combined LDL and expectation regression into a unified structure to ease the disparity between the training and evaluation stages. The proposed approach efficiently enhanced the performance of the earlier DLDL on both prediction error and inference speed for age classification. The approach's effectiveness was validated for real and apparent age estimation tasks using MORPH-II, LAP-2015, and LAP-2016 datasets.

Duan et al. (2018a) developed a robust age estimator which employed an ensemble structure: CNN2ELM, which includes CNN and extreme learning machine (ELM). The model updated the work presented in Duan et al. (2018b) and has a three-level approach, including feature extraction, age grouping using an ELM classifier, and age estimation with an ELM regressor. The model initially pre-trained on the ImageNet dataset was fine-tuned on the IMDb-WIKI and MORPH-II datasets. The experimental analysis performed on the LAP-2016 dataset outperformed the existing state-of-the-art age estimation models and achieved an ϵ-error of 0.3679.

The deep expectation model based on VGG-16 architecture was proposed (Rothe et al., 2018). The approach is a solution to real and apparent age estimation from a single face image without the use of facial landmarks. The DEX model was pre-trained on both ImageNet and IMDb-WIKI datasets to achieve better performance. They evaluated their method on three standard datasets, MORPH-II, FG-NET, and LAP-2015, and obtained state-of-the-art results for both real and apparent age estimations. The approach achieved an MAE of 3.09 (FG-NET), 2.68 (MORPH-II), and 6.521 (CACD), an ϵ-error of 0.2650 (LAP-2015), and 64.0% (Exact), and 96.6% (1-off) accuracy on the Adience dataset outperformed the existing state-of-the-art age estimation methods. It recorded an ϵ-error of 0.3679 on the LAP-2016 dataset.

Li et al. (2019) proposed a CNN-based technique called BridgeNet for real and apparent age estimation. The proposed model comprises two components: local regressors and gating networks that can jointly be learned end-to-end. The first component (local regressors) addressed heterogeneous data by partitioning the data space. In contrast, the second one (gating networks) employed a bridge-tree structure that learns the continuity-aware weights used by the local regressors. Experimental results on the MORPH II, FG-NET, and Chalearn LAP-2015 datasets show the effectiveness of the BridgeNet CNN in outperforming the state-of-the-art methods.

Liu et al. (2019) developed a method that is an extension of their work in Liu et al. (2018). The work is an end-to-end ordinal deep learning (ODL) framework, including two ordinal regression loss functions; square loss and cross-entropy loss. The proposed ranking-based ordinal deep feature learning (ODFL) method learns features needed for face representation directly from raw image pixels and then independently learns the procedures of feature extraction and apparent age estimation. The work was evaluated on state-of-the-art face aging datasets and achieved superior performance compared to state-of-the-art methods in apparent age estimation.

The work presented by Dagher and Barbara (2021) employed transfer learning from four pre-trained CNNs models to develop a facial age estimation model of human faces. The authors aimed to find the optimum age gap and to achieve high age estimation accuracy. The model was trained and evaluated on the FG-NET + MORPH datasets. These datasets were deemed suitable because it has varieties of age range from 0 to 77 years. The proposed model achieved an MAE of 2.94 and 2.97 on FGNET and MORPH datasets, respectively.

Recently, Zhao et al. (2022) proposed an adaptive mean-residue loss effective for facial age estimation. The proposed mean loss can penalize the age probabilities between the estimated age distribution's mean and the apparent age. Experiments for the model were performed on popular facial datasets (FGNET and CLAP2016) and evaluated using the MAE and ϵ-error to achieve 3.61 and 0.3882, respectively. The experimental results show superior performance on both datasets when applied to state-of-the-art models (VGG-16, ResNet-50) compared to the existing mean-variance loss methods. The authors conclude with some recommendations that can improve the performance of their model.

A deep learning model that comprises five convolutional layers and three fully-connected layers trained from scratch was developed and presented in Kjærran et al. (2021) for individual age estimation from facial images. The model was trained on three benchmarks (APPA, UTK, and IMDB) datasets and evaluated on separate held-out data and the Adience benchmark datasets. The experimental results show an inferior performance on the Adience dataset compared to existing models on the same dataset. In contrast, improved performance was obtained when the model was evaluated on the held-out dataset to achieve exact accuracy of 0.304% and one-off accuracy of 0.463%.

Deep learning models applied to facial age estimation tasks and the different data modalities employed in studying aging were presented in Ashiqur Rahman et al. (2021). The present study presents four broad classes of measures for quantifying algorithms' performances concerning biological age estimation. Based on the findings, the direction for the future endeavor in the apparent age estimation research was identified with significant potential for improvement in understanding the individual's health status with respect to body shape, blood samples, and physical activities.

A hybrid facial age estimation method using the Gabor feature fusion with an atomic search algorithm for feature selection was proposed by Lu et al. (2022). Gabor filter with five scales and eight directions was first used to extract facial age features and then employed a histogram to carry out coding and fusion for the indices in each direction of the Gabor. An algorithm called the chaotic improved atom search optimization with simulated annealing (CIASO-SA) was then presented to improve the accuracy and the number of feature selection that is more adaptive to solving optimization problems in high dimensions. Experimental results found that Gabor achieved the best results on the 48 x 48 image resolutions to obtain one-off accuracy of 85.9%.

Deng et al. (2021) proposed a multifeatured learning and fusion method for age estimation. Three subnetworks were employed to learn age, gender, and race information. The race and gender information and the age features were concatenated to form a robust feature extraction for age estimation. These features were then converted into exact age using the regression ranking age feature estimator. Three popular benchmark datasets (MORPH2, FGNET, and LAP) were used to experiment and validate the performance efficiency of the model. The proposed model achieved an MAE of 2.47%, 2.59%, and 2.67%, respectively, for the three datasets, compared to existing methods. The model is also suitable for mobile device deployment for age estimation due to memory compactness of 20 MB only.

A gender-specific facial age estimation system was proposed by Raman et al. (2022) to classify gender images and estimate age. The system is composed of two separate models. One of the components is meant to classify the facial images into males and females separately. The other component is made up of models that were trained independently on the male and female images. The models were trained on and evaluated on the UTK-Face dataset, while cross-validation was performed with the FGNET dataset. The experimental results report 90.86% of accuracy on the female-specific model and 89.21% of accuracy for the male-specific model.

## 5. Apparent age estimation datasets

We briefly highlighted three popular facial aging datasets that were widely employed in the research of apparent age estimation; Chalearn LAP-2015, Chalearn LAP-2016, and APPA-REAL datasets.

**ChaLearn LAP-2015 dataset**. Escalera et al. (2015) was collected for the purpose of ChaLearn LAP challenge competition 2015 edition. It is the first dataset on apparent age estimation. The dataset comprised 4,699 images, with each image labeled by at least ten different users. LAP-2015 dataset is divided into three sets: 2,476 (training), 1,136 (validation), and 1,087 (testing).

**ChaLearn LAP-2016 dataset**. Escalera et al. (2016), an extension of the LAP-2015 dataset, consists of 7,591 face images collectively labeled by different human annotators. The dataset is divided into three sets: 4,113 training, 1,500 validation, and 1,978 testing images. The testing set labels are separated from the other sets but with similar age distribution.

**APPA-REAL dataset**. Agustsson et al. (2017) is the first state-of-the-art database with both real and apparent age annotations. The images are collected using a labeling application, crowd-sourcing data collection, data from the AgeGuess platform, and with the assistance of Amazon Mechanical Turk (AMT) workers. APPA-REAL database contains a total of 7,591 images with an age range between 0 and 95 of images of subjects taken under different conditions.

**Figure 3:**
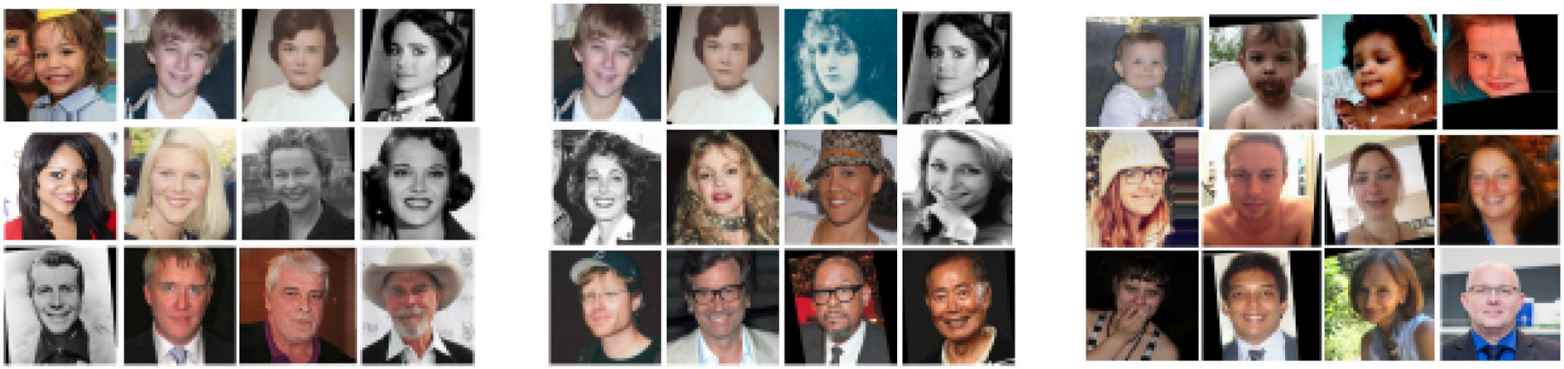
Sample images from LAP-2015, LAP-2016, and APPA-REAL datasets. LAP-2015 and LAP-2016 datasets were collected purposely for the Chalearn looking at people (LAP) competition in 2015 and 2016, respectively. It contains facial images with apparent ages. APPA-REAL dataset, on the other hand, was introduced by Agustsson et al. (2017) and contained images with both real and apparent age annotations.

**Figure 4:**
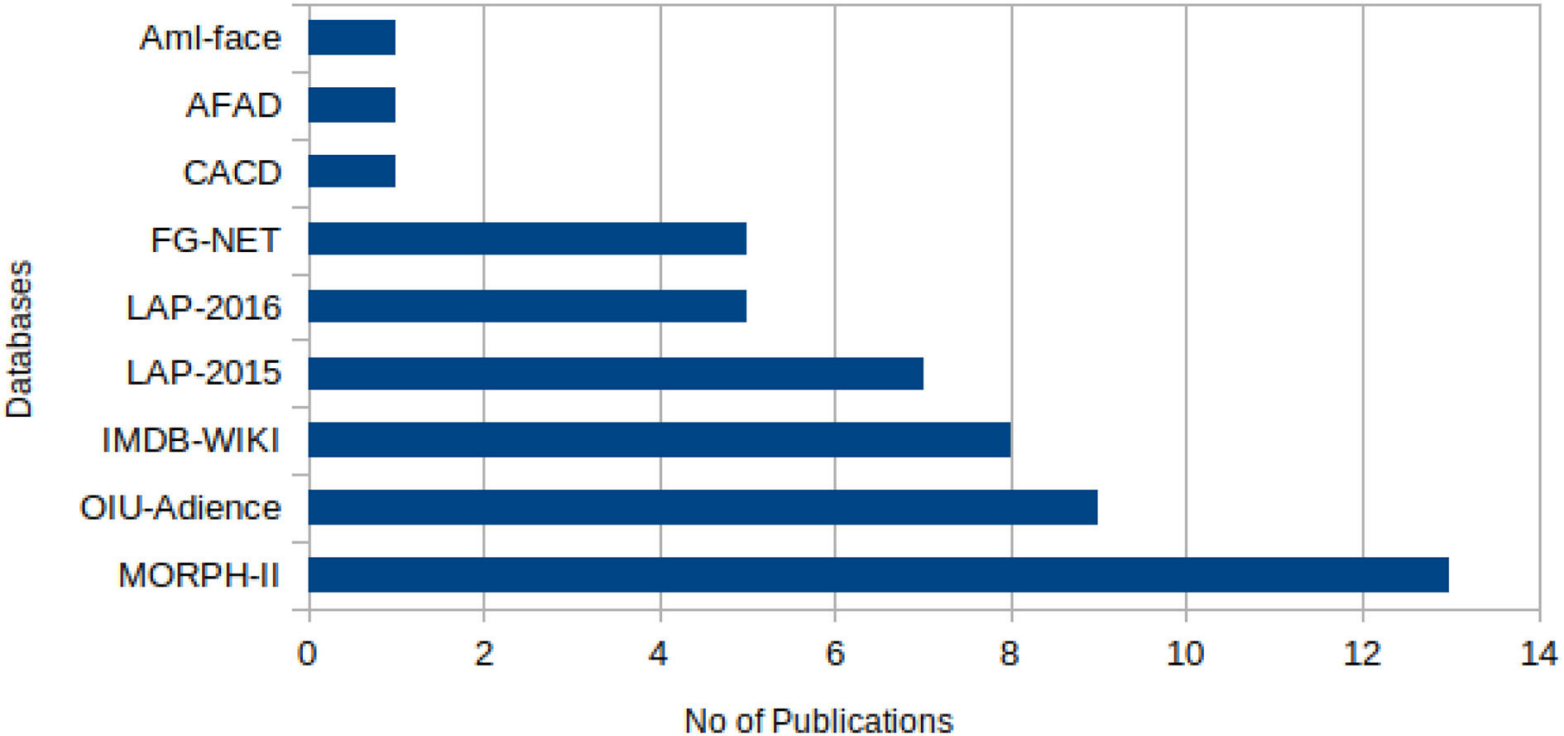
Facial aging datasets by the number of publications (CNN). MORPH-II dataset has the highest number of usage in the evaluation of real-age estimation models. LAP-2015 and LAP-2016 datasets are the literature's most common publicly-available apparent age estimation datasets.

**Figure 5:**
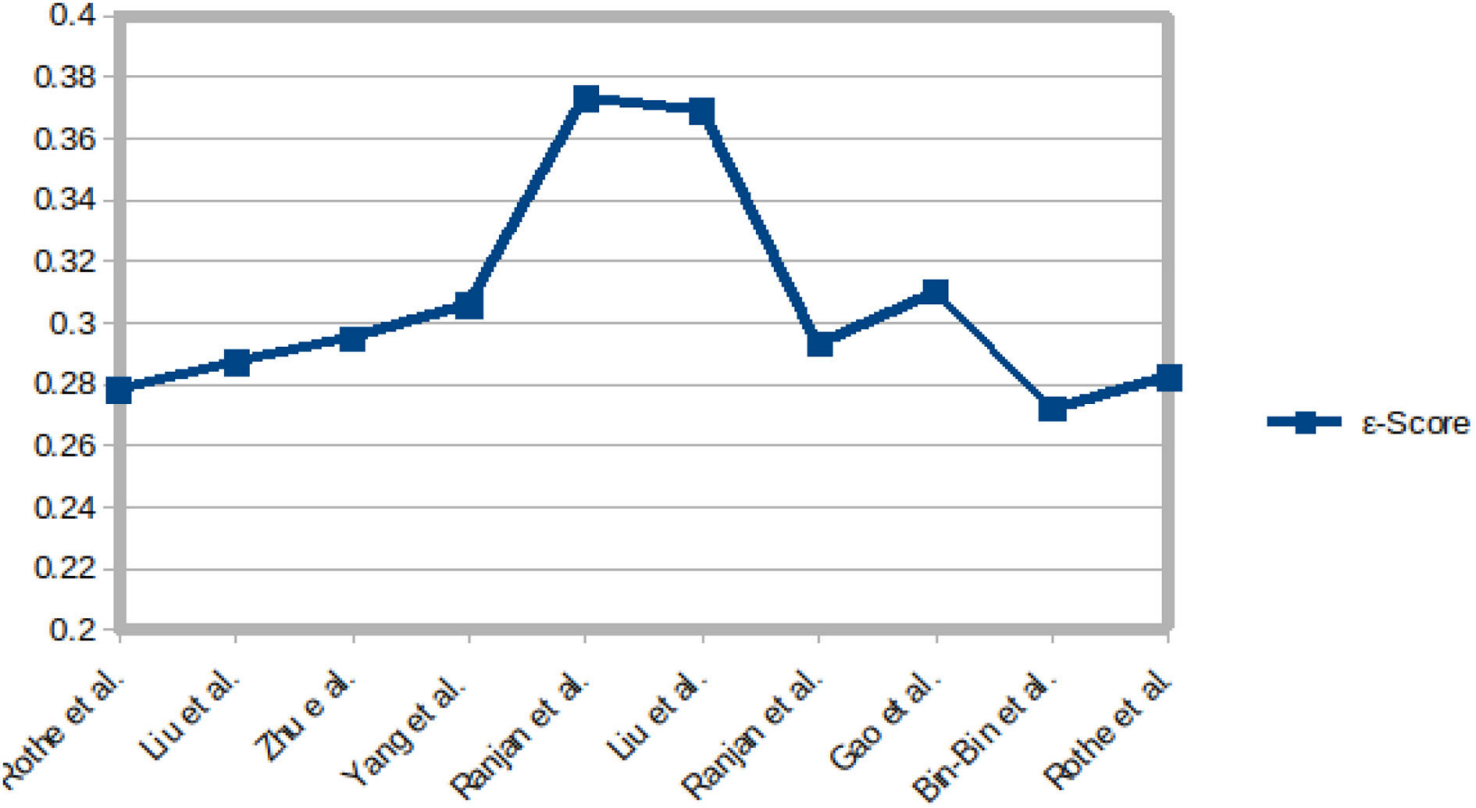
Performance, in terms of ϵ-error, achieved by some authors on the Chalearn LAP-2015 dataset.

The sample images from these datasets are presented in Figure 3, while sample size, subjects size, and the age range information of those datasets are presented in Table 3. Figure 4 shows facial aging datasets by the number of publications, while Figure 5 shows the performance in terms of ϵ-error achieved by authors on the Chalearn LAP 2015 dataset.

**Table 3:** A summary of apparent age estimation databases.

**Database**	**#Faces**	**#Subj**.	**Range**	**Age type**	**Year**	**In-the-wild?**
ChaLearn (Escalera et al., 2015)	4,699	-	0–100	Apparent	2015	yes
ChaLearn (Escalera et al., 2016)	7,591	-	-	Apparent	2016	yes
APPA-REAL (Agustsson et al., 2017)	7,591	7,000+	0–95	Real and Apparent	2017	yes

## 6. Performance evaluation metrics for the state-of-the-art methods in apparent age estimation

The standard evaluating metrics used for facial age estimation are MAE (Onifade and Akinyemi, 2014), cummulative score (CS) (Lin et al., 2012), accuracy (exact and 1-off) (Levi and Hassncer, 2015), and normal score (ϵ-error) (Duan et al., 2018a). MAE, CS, and accuracy are commonly employed for real age estimation, while MAE and ϵ-error are the common evaluation metrics for apparent age estimation. MAE is defined as the average of the absolute errors between the estimated ages and the ground truth, and CS measures the performance of an estimator when the training data has images at nearly every age; exact accuracy calculates the percentage of face images that were classified into correct age and gender; the ratio of the accurate predictions to the total number of the ground-truth label while one-off accuracy measures whether the ground-truth class label matches the predicted class label or if the ground-truth label exists in the two adjacent bins.

Mean Absolute Error is defined mathematically by:


(1) $$\begin{array}{l} MAE=\overset{N}{\underset{k=1}{\sum }}\frac{\left|l_{k}-l_{k}^{*}\right|}{N} \end{array}$$

where *l*_*k*_ : the estimated age.$l_{k}^{*}$ : the ground truth age for the test image k.*N*: the total number of test images.

Normal (ϵ)-error metric was employed for the purpose of the Chalearn LAP competition in 2015. It calculates the error between an estimated value and the average labeled age. ϵ-error is calculated as follows:


(2) $$\begin{array}{l} \epsilon =1-e^{-\frac{{\left(x-\sigma \right)}^{2}}{2\mu^{2}}} \end{array}$$

where x: the estimated age.σ: apparent age label provided for a given face image.μ: standard deviation of all estimated ages for the given face image.

ϵ-error not only calculates the error between the estimated value x and the averaging labeled age σ but also considers the standard deviation of μ. The final ϵ-error result is the average overall prediction, and obviously, the lower the ϵ-error, the better the estimator's performance.

**Figure 6:**
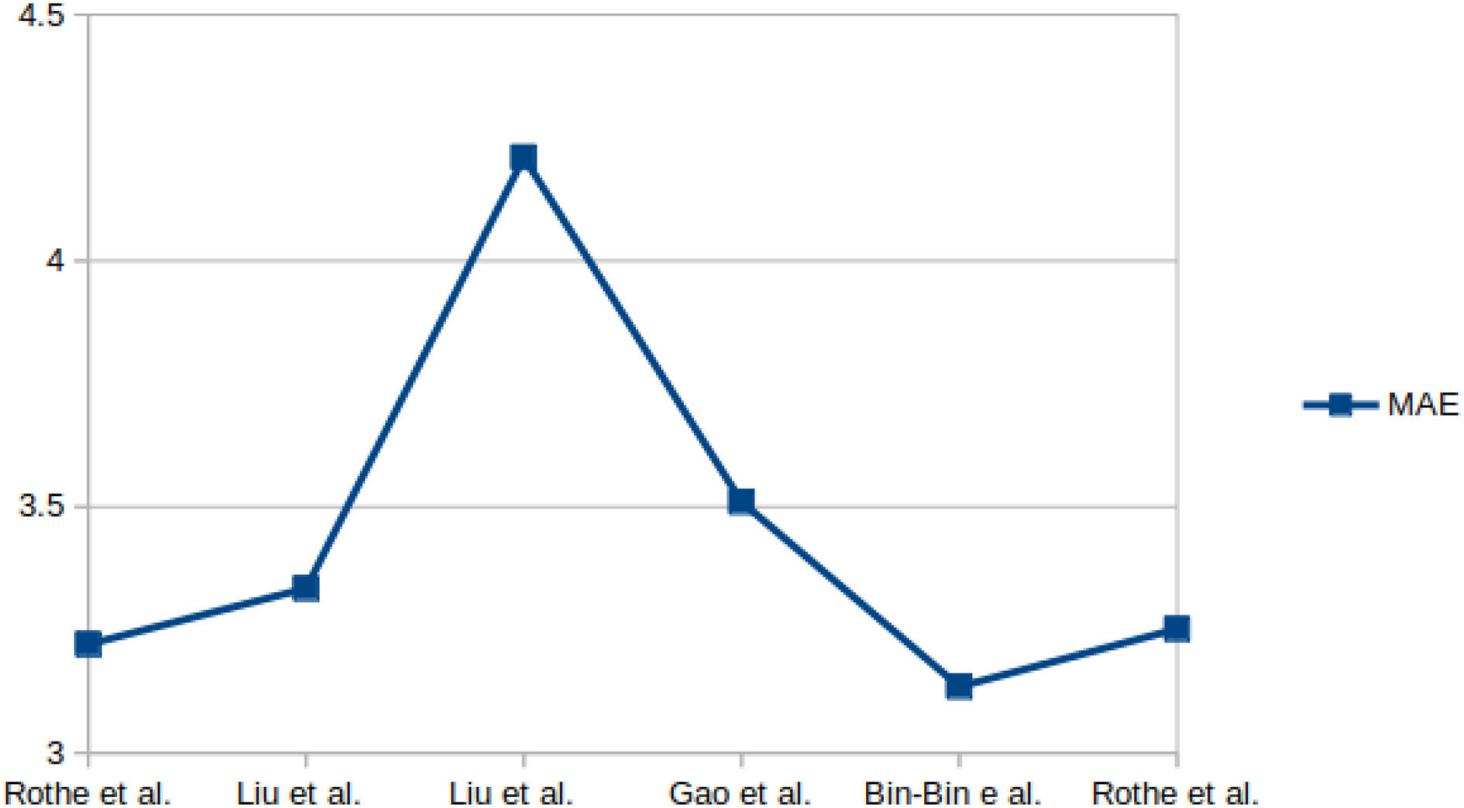
Performance, in terms of MAE, achieved by some authors on the Chalearn LAP-2015 dataset.

**Figure 7:**
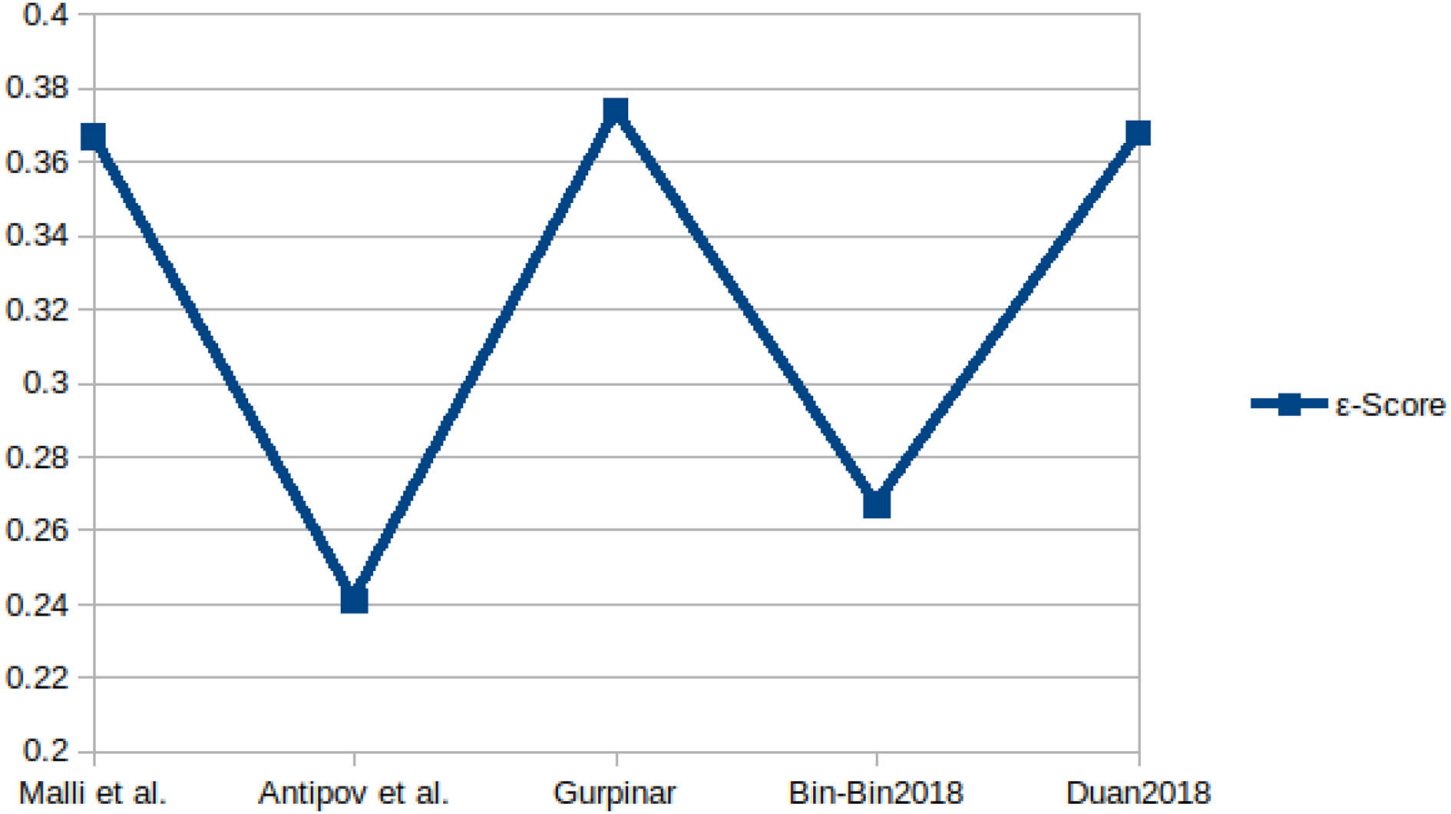
Performance, in terms of ϵ-error, achieved by some authors on the Chalearn LAP 2016 dataset.

Figures 6, 7 present the MAE and the ϵ-error performance reported by authors on the Chalearn LAP 2015 and Chalearn LAP 2016 datasets.

## 7. Discussion

In recent times, a study in apparent age estimation has progressed steadily in terms of performance. From the literature presented above, it was clear that no concise conclusion can be made on the best type of algorithm for the estimation task. In Table 1, we present a description of each of these algorithms employed by researchers, highlighting their strengths and weaknesses; this will help to choose the right algorithm for future work.

As presented in Figure 4, it was observed that the MORPH-II dataset had been far more used for real age estimation experiments than any other datasets, and this is probably due to the more significant number of facial images, which helps algorithms to learn the many individual aging traits better. However, LAP-2015, LAP-2016, and APPA-REAL are the three publicly-available facial aging datasets with apparent age labels for apparent age estimation. In order to avoid biased, considering the peculiarities of each dataset, our analysis will be based on these three datasets. As such, we summarized in Table 2, the performance of state-of-the-art algorithms showing a clear distinction between which result is best so far on each of these datasets for apparent age estimation when evaluated using MAE and ϵ-error metrics.

Consequently, significant observations from earlier research work in the estimation help make some justifiable conclusions. Hence, we highlighted those observations stating some of the points which we deem very important:

- It was observed that the image processing method employed for face detection, facial landmark, and face alignment has an impact on the performance of an apparent age estimator.
- It was also important to assert that the performance of learning algorithms was determined by many factors, among which are the size and label distribution of the employed dataset, the degree of image variability, etc. The deep learning algorithms perform differently on different datasets, most likely due to the peculiarities of each dataset.
- Data augmentation improves the performance of age estimation models, especially on unevenly distributed not-too large datasets.
- We also observed from the literature that models pre-trained on large-scale datasets before fine-tuning on the original dataset performed better than training the model on just the original dataset.
- From this review, we observed that MC algorithms had been the most popularly used individual algorithm for apparent age estimation on the mentioned datasets.
- We also observed that ranking and DLDL are the most suitable algorithms for the estimation when the label distribution of the dataset is uneven.

For apparent age estimation on LAP-2015, a hybrid algorithm (LDL and expectation regression) showed the best performance on MAE and ϵ-error metrics (Figures 5, 6). However, on the LAP-2016 dataset (in Figure 7), a hybridized algorithm combining label distribution and classification algorithm also presented the best performance. The choice of any of these algorithms continues to be debated in the facial aging research community, which is evident in Table 2. This demonstrates that no approach may be individually suitable for apparent age estimation; instead, an MC, DLDL, or hybrid (the combination of DLDL with another algorithm) seem more effective and consistent.

## 8. Conclusion and future directions

This work presents a comprehensive review and the suitability of modern algorithms for apparent age estimation focusing on those algorithms that are most popular and those that appear to have been the most successful. In this review, we based our evaluations on LAP- 2015, LAP-2016, and APPA-REAL, the most popularly-used publicly available facial aging datasets for apparent age estimation. Apparent age estimation, estimate “how old the person looks like”? A hybridized algorithm showed the best performance on LAP-2015 and LAP-2016 datasets. From this study, we could deduce that MC was the most popularly used individual algorithm. We also asserted that the performance of these age estimation algorithms is mostly influenced, not only by choice of the approach but also by some other factors, among which are the method of image pre-processing applied, size and the distributions of the employed datasets, etc.

However, many encouraging future directions in apparent age estimations may improve performance. Huge datasets with apparent age label annotation rather than real age will help improve the research accuracy of apparent age estimation. Also, it is necessary to investigate further study that focuses more on predicting apparent age (how old does the person look?) rather than a person's biological age to enhance the practical and real-world applications of this research.

## Author contributions

All authors listed have made a substantial, direct, and intellectual contribution to the work and approved it for publication.

## Conflict of interest

The authors declare that the research was conducted in the absence of any commercial or financial relationships that could be construed as a potential conflict of interest.

## Publisher's note

All claims expressed in this article are solely those of the authors and do not necessarily represent those of their affiliated organizations, or those of the publisher, the editors and the reviewers. Any product that may be evaluated in this article, or claim that may be made by its manufacturer, is not guaranteed or endorsed by the publisher.
